# Obesity, Health Disparities, and Prevention Paradigms: Hard Questions and Hard Choices

**Published:** 2005-09-15

**Authors:** Shiriki Kumanyika

**Affiliations:** Department of Biostatistics and Epidemiology, University of Pennsylvania School of Medicine, CCEB

## Introduction

In public health, because of our commitment to advocacy, there is always the danger of becoming a *believer *— becoming so convinced about the issues one is pursuing that it becomes difficult to consider new information objectively. Although a degree of subjectivity is inherent in all scientific inquiry, one who believes uncritically risks missing opportunities to progress based on new insights, or, worse, one may introduce bias into inquiry — selectively evaluating data or studying the question in a way that favors a certain answer. I find myself increasingly preoccupied with these considerations as my work on obesity and minority health takes me into policy arenas where I must sometimes advocate for and defend a particular position. While issues of objectivity are relevant to public health generally, I find them particularly pertinent to obesity, health disparities, and prevention because — for different reasons — all three of these topics can be polarizing and politically charged.

The politically charged nature of obesity research in an ethnic context first drew my attention at a 1984 National Institutes of Health (NIH) workshop I attended. A black woman researcher vociferously accused a workshop presenter (a white man) of racism and stereotyping when he interpreted a slide as showing the "well-known finding" that black women have a greater prevalence of obesity than white women. As an onlooker, I realized that the presenter might have been right, but that the ability to openly study, discuss, and ultimately solve the problem of obesity among black women was constrained by the sensitivities associated with obesity and the politics associated with being black in America. I decided then to take this issue on (1). As a black woman researcher, I would be in a politically safer and perhaps even strategic position to press for more attention to these issues. The key research questions were obvious — what was the reason for the high prevalence, and what could be done about it? However, these obvious questions have not had obvious answers, even to date, and the topic is still difficult to talk about without getting into trouble. But I press on, treating the topic with caution and respect, because I continue to be distressed, personally, at seeing my own reference group so affected. To guard against the danger of being too much of a believer, I sometimes, as here, find it useful to play the contrarian to my own position on the importance of obesity prevention in the African American community. Through this process of self-challenge, I push back against my own subjectivity to gain a clearer understanding of what the issues are in African American health and how best to approach them from a holistic perspective.

## Confronting the Issue

The obesity prevalence data for black women are, by now, all too familiar. Seventy-seven percent of black women are in the overweight or obese (defined as having a body mass index [BMI] ≥25 kg/m^2^) range, and 50% are in the obese range (BMI ≥30) (2). The severity of obesity among black women is also greater than average when judged by the 15% who have a BMI of ≥40 (the Class III or "extremely" obese range) (2). The variation in obesity with socioeconomic status (SES) must be considered when comparing blacks and whites, but the higher obesity prevalence in black than white women is seen at all levels of typical SES indicators such as education and income (3). The problem is not confined to adults. Among black girls, the high prevalence of obesity is of relatively recent onset but seems to have caught up with and passed the prevalence of obesity among white girls (4,5).

## The Political Nature of Health Disparities

When then U.S. Secretary of Health and Human Services Margaret Heckler released the *Report of the Secretary's Task Force on Black and Minority Health* in 1985, obesity was among the modifiable risk factors associated with leading causes of "excess deaths" (6). Excess deaths were defined as the numbers of deaths observed in specific minority populations that were in excess of those that would be expected on the basis of age- and sex-adjusted data for the majority white population (6). Six causes of excess deaths were identified — cardiovascular diseases (CVDs), cancers, homicide–suicide–intentional injuries, diabetes, infant mortality, and cirrhosis of the liver. Obesity was specifically linked to CVD and diabetes. 

Who or what is to blame for these disparities is a political issue. Do the disparities reflect a failing of society to provide everyone with an equal opportunity to be healthy? Do they reflect institutionalized patterns whereby certain racial/ethnic groups are systematically left open to abuse (e.g., structural violence), as argued by Paul Farmer (7)? Or are the excess deaths thought to be attributable to inherent defects (e.g., eugenics) or moral/behavioral failings of the affected populations — too much risk due to too much "bad behavior"? When Secretary Heckler released the task force report, the message was framed in terms of individually modifiable risk factors (i.e., more toward the "bad behavior" than the "bad society" type of explanation). Here was the clarion call for minority populations to change *their* behavior, to modify *their* risk factors that would cause *their* disparities to disappear.

However, the pervasiveness of disparities affecting diverse racial/ethnic minority populations across the spectrum of health outcomes speaks loudly to the point that structural factors are also involved. It is not simply *their behavior* that needs changing. The dialectic around health disparities continues to focus on equity and social justice and on the fallacy of interpreting as genetic the systemic, biologically relevant, and transmissible health effects of responses to institutionalized racism and social disadvantage (8,9). From a minority health advocacy perspective, the disparities are the hard evidence of decades of oppression and mistreatment. The greater the disparity, the more legitimate the demand for political focus and funding from Washington. Each minority population is left to make sure that the particular disparities relevant to their situation receive sufficient attention — a somewhat depressing competition for who can be seen to have the worst health profiles. The irony in this scenario is that to succeed in reducing the disparities is to risk falling off of the radar screen.

## Asking the Hard Questions

There have always been naysayers about the importance of obesity to health. Nevertheless, I was shocked to hear Paul Campos, author of *The Obesity Myth* (10), invoke data on the lack of association between obesity and mortality among black women to buttress his case that the current level of public health attention to obesity is misguided (11). I knew the data to which he referred and strongly disagreed with his interpretation. Like the aforementioned confrontation at the NIH workshop in 1984, this moment led me to consider the potential validity of Campos' argument and to challenge my own preconceptions. Taking up the gauntlet thrown down by Campos and incorporating my continuing concerns, I have framed the four hard questions that follow.

### 1) Do we really know that obesity poses a risk to health in African Americans?

The answer depends in part on how one defines health risks (i.e., in terms of mortality or both mortality and morbidity) and how the data are analyzed. The higher mortality of African Americans from obesity-related conditions such as CVD, diabetes, and certain types of cancer (12) does not necessarily mean that obesity is a key factor driving these rates. Obesity could be "present but not guilty," since these diseases have multifactorial causation, and one might readily conclude this from data in which mortality rates for African Americans at the lean and obese ends of the BMI continuum are compared (13). The slope is often surprisingly flat along most or all of the BMI range — which was the point made by Campos. The expectation, based on data for whites, is to see increasing mortality with increasing BMI. However, the association between BMI and mortality is weaker in blacks than in whites in most or all relevant data sets — for a variety of possible reasons that have been discussed in a thoughtful review by June Stevens (13). Similar to her, my sense has been that comparisons of relative risks across ethnic groups with grossly different mortality profiles can be misleading. Mortality rates are higher in African Americans than in whites across the entire BMI distribution, and the mix of causes of death differs by ethnicity. Furthermore, death rates reflect not only disease incidence but also all of the other variables that influence when death occurs among people who have a disease — timing of diagnosis, the access to and quality of treatment, adherence to treatment, comorbid conditions, non-disease-related causes of death, and other less well-defined social and environmental factors that are reflected in death rates and that differ between blacks and whites. My concern is that there is an over-reliance on associations between obesity and mortality without also considering effects of obesity on disease and disability.

Selection of indicators for comparing BMI-related risks in ethnic groups with different disease and death rates is not straightforward (14). As with comparisons of the obesity–mortality associations across age groups (15), the risk in the obese relative to the lean is influenced by the rates in the lean reference group. Stevens illustrates this by noting that comparisons based on the rate difference (i.e., the rate in those with high BMI minus the rate in those with low BMI) give a different impression. Such comparisons show the absolute numbers of people affected in the groups being compared (which are higher in the groups with higher overall death rates) (Table) (13). Dr Stevens comments: "Most notably, it appears that the relatively smaller increase in the obesity-associated relative rate of mortality in African Americans compared with whites should not be interpreted to mean that obese African Americans have a lower risk of death than obese whites" (13). She points out that the largest difference in death rates between black and white women is not in the obese group but rather in the normal weight group (Table). However, in spite of the lower relative risk, it is difficult to conclude that obesity and, presumably, obesity-related morbidities in black women are making no contribution to their mortality.   

If one concedes that disease and disability are an important part of the picture, then there is strong evidence that obesity can worsen the health of both African American women and men. This evidence comes from studies that show increased rates of development of disease and disability in those with high BMI levels compared with leaner counterparts (16-19). Furthermore, some randomized controlled trials have reported equivalent or better improvements in risk factors or decreases in rates of disease occurrence in association with weight loss in African Americans compared with whites (20-23). So, I conclude that we are on solid ground in considering obesity as a health problem in African American communities.

### 2) Will the increased focus on obesity further stigmatize African Americans?

Controversial images of overweight black women have been with us for decades: depictions of Aunt Jemima (24) or the role of Mammy in *Gone With the Wind*. Kathleen LeBesco, author of a recent book about cultural and political aspects of attitudes toward obese people, argues that the current slimness craze is rooted in an effort to stigmatize groups such as African American and Mexican American women (25). Although I disagree with LeBesco's line of reasoning, her assertions are a grim reminder of the combined impact of stigma related to race and weight.

This is truly a catch-22. If nothing is said or done about obesity among black women, the problem and its health consequences cannot be addressed. On the other hand, when attention is drawn to the high prevalence of obesity among black women, denigrating stereotypes of black women that are already deeply embedded in American culture (24) may be enhanced. I am not sure how we can work around this issue.

### 3) What can we really promise with respect to the benefits of weight loss?

The promise of health benefits from weight loss cannot be fulfilled without effective weight loss programs. We still know very little about how to control weight over the long term, and we know even less about how to control weight among African Americans and other ethnic minority populations (26,27). Studies from which a direct comparison can be made between weight loss results for black and white participants suggest that the best treatments do not work as well in blacks as in whites (27). Evidence about biological explanations for this difficulty with losing weight is not convincing (28), whereas explanations based on environmental and behavioral determinants are convincing, but these factors are not easy to change (27). Culturally adapted weight loss programs, where they have been evaluated, have not met with overwhelming success, although such programs may be well received and culturally salient (27). There might be effective programs in communities, but few community programs have been evaluated. 

Studies to identify effective strategies for obesity prevention and treatment in black and other minority communities are just emerging. The most we can promise for the time being is willingness to work with communities in the development and evaluation of potentially effective programs. Waiting with hands folded because we do not have the perfect solution is not an option, but frustrating communities with ineffective programs is also not a good idea. 

### 4) Is obesity really a high priority in the face of other health disparities?

Obesity and obesity-related conditions are clearly not the only important health disparities occurring within the complicated and changing societal context affecting African American communities (12,29,30). Those of us in the nutrition, physical activity, and obesity fields must face the question of whether, for the overall good of the black community, for example, some of the resources devoted to obesity would be more appropriately placed elsewhere. Take data on women's health, for example. While it is true that heart disease, stroke, and diabetes — all of which are obesity related — are the three leading causes of death for black women and affect large numbers of black women, the more dramatic disparities relative to white women are in conditions that threaten black women in their prime: the risk of developing acquired immunodeficiency syndrome (AIDS) (incidence ratio of about 20 to 1 for blacks vs whites), maternal mortality, or homicide (both with a ratio of about 4 to 1 for blacks vs whites) (31). The black–white gap in infant mortality, with a ratio of about 2 to 1, also persists as a continuing reminder of the ethnic differences in life chances from conception onward. There is no obvious answer to the question of which of these problems is the most important. They are all important.

## Facing the Hard Choices

So far, I have been taking the view of an African American researcher. When I view these issues as a member of the African American community at large, the hard questions become a set of hard choices that black communities face. Do African American community advocates and community members set priorities based on their perceptions of social injustice, that is, on statistics about how bad things are relative to whites, or on the immediate goal of avoiding day-to-day pain and suffering? Does it sound ridiculous to tell people about the risk of death from obesity-related diseases somewhere down the road when there are frequent reminders of the risk of death right outside? People in communities should not have to choose among various problems, all of which are pressing at some level, simply because we scientists and professionals have bureaucratized ourselves along problem-specific lines. In reality, multiple conditions or health outcomes coaffect the same individuals, families, and communities and have common underlying causes. It is we who must find ways to address multiple problems in an integrated manner as they are experienced in communities. 

Another difficult choice for communities is whether or not to attack problems as they appear on the surface — the symptoms — without demanding attention to the underlying ills that continue to erode communities and the quality of the lives therein (32). Overeating, for example, is a complex behavior that contributes to the obesity problem among black women (33,34). Superficially this problem can be approached with counseling about how to eat less, but black women may want help in addressing the underlying factors that promote overeating: the excess availability of high-calorie foods, particularly in segregated neighborhoods that have a deficit of supermarkets and a surfeit of fast food restaurants (35-37); food insecurity (38); and the need to cope with stresses stemming from racism (8,9). Worse, the superficial solutions, such as behavioral counseling without environmental amelioration, may very well create guilt and frustration associated with knowing, but somehow not doing, what is needed for weight control.

Whether to risk damage to a positive self-image by making body size issues more a problem for black women is an additional dilemma. Populations with a history of oppression have, of necessity, honed their ability to be self-accepting to a fine art in order to survive, to buffer mistreatment and derogatory images from outside the community (e.g., the idea that maybe society does not love us, but we can love ourselves). Some obese black women may have a strong self-image that transcends weight issues and is somewhat resistant to the mainstream stigmatization of weight. Emphasizing weight issues within African American communities, in which nearly 80% of women would be targeted as overweight or obese, would raise the potential for harmful effects on self-esteem. Attitudes among African Americans and other populations with a history of economic stress and deprivation include some that equate being heavier with being healthier relative to thin people. Thin people may be seen as wasting away due to illness or addictions, leading to attitudes that are less negative about excess weight than in the mainstream (39,40). Spiritually, African Americans may be counseled to "be satisfied with what God gave you" or, practically, to "make the best of what you have" (41). Personal acquiescence is also a survival skill among the oppressed. 

The call for culturally adapted programs raises the issue of cultural relativism. A purely relativistic approach would be to attempt to formulate weight control programs and interventions in the context of the cultural perspectives in African American communities, under the assumption that these cultural perspectives are not only valid but also dominant considerations for achieving salience and effectiveness of programs that address eating behaviors and physical activity lifestyles. The extreme opposite would be the "cultural imposition" (42) of a mainstream perspective about weight control onto African American communities. My current hypothesis (or hope) is that there may be some effective blend of strong, culturally valid programmatic conceptualizations or adaptations with strong behavioral change strategies from mainstream programs, but I have not yet had time to put this to the test. 

Finally, there is the question of how we prioritize the areas of obesity prevention and weight stabilization. Weight stabilization makes sense as the first step on the path toward decreasing obesity prevalence over the longer term and thereby decreasing the incidence and consequences of CVD and diabetes. However, the longer we wait to implement preventive strategies, the longer the continuing influx of people into the overweight and obese categories. Ultimately, when African American communities consider the potential reduction in the health burden and costs as well as improvements in quality of life that effective obesity treatment might bring, simply holding the line with the current high rates of overweight and obesity will not suffice.

## Prevention Paradigms

A part of the uncertainty in moving forward with preventive strategies may be the somewhat politically charged debate about the best choice of prevention paradigms (43,44). One paradigm conceives of the progression to obesity on a continuum where those who are not yet obese but who are above normal weight are *pre-obese* (i.e., in the range below the clinical horizon or threshold that defines obesity). The term *pre-obesity* labels a substantial proportion of the population, and particularly of ethnic minority populations, as having a condition that requires medical treatment. Other paradigms take a health promotion or population health approach, focusing on whole populations and advocating approaches whose goal is to shift the entire BMI distribution to have a lower mean (45). Population health approaches emphasize the need for social change and political will to effect improvement in the environments for achieving or maintaining energy balance (e.g., policies and programs that make lower calorie food options or smaller food portions more available and affordable and that increase opportunities for being physically active while decreasing sedentary time). Population- focused approaches are also termed "upstream" approaches because the levers involved are several layers removed from "downstream" — the level of individual choices — where the problems become visible (45).

The dominant paradigm in the United States has been the treatment of the individual. There is a lot of support for and often a high comfort level with individually oriented approaches. They are closer to what many in the health care field have been trained to do, which is to treat disease, and they are easier to evaluate with familiar research designs such as randomized controlled trials. These approaches are also politically safe and do not directly challenge the commercial vested interests with a stake in the current obesogenic environment. They do not raise the particularly American sensitivity for possible infringement on choice that might accrue from large-scale environmental or policy changes. Furthermore, the individually oriented approaches emphasize "personal responsibility" as the first principle of solving societal problems, the view that Dr Heckler espoused when she released the report of the Task Force on Black and Minority Health in 1985. Of course, this is also the view associated with "blaming the victim."

I agree that individuals should be held accountable for their actions. However, all actions happen in context. When the environment is so heavily loaded toward fattening the population, the range of eating and physical activity choices available to consumers is slanted in an undesirable direction. It is very consumer unfriendly, and perhaps even cruel, to put the burden entirely on consumers to foster the needed shift in the demand-supply curves that relate to obesity, particularly for consumers in disadvantaged communities where the range of choices may be especially unfavorable and where most of the economic and political forces involved are far beyond their personal or aggregate control. I strongly favor a population-oriented paradigm that selectively incorporates programs for individuals.

## Conclusion

Looking back at the questions I posed, I say with confidence that obesity is a serious health problem in the black community. The need for action is even more compelling when the implications of the rise of obesity in African American children are factored in. However, with respect to other questions, the answers become a lot less obvious. Great care must be taken in any public campaign on the obesity issue not to stigmatize anyone, especially children, for whom the self-esteem and long-term attitudinal and behavioral issues are even more complicated than for adults, and especially in populations who face daily onslaughts to their self-esteem related to prejudice and discrimination. As for interventions, I think the message is clear that we need to either figure out ways to generate effective obesity interventions in the black community and in other communities of color where excess risk is observed, or we should get out of the business. We have to offer more, much more, than we do now.

While I personally think obesity deserves a high priority in African American communities, I would leave the decision about how to prioritize to the communities themselves. Professionally, we should work more holistically with communities so that they don't have to choose between obesity and other problems, especially since, from a practical perspective, the more immediate issues always win out when people have limited means and no real choice. It would also help to identify subgroups at high obesity-related health risk from a phenotypic perspective. Not much has been done in this arena. Being able to identify which population subgroups are most likely to develop the health problems associated with obesity may become important when working with communities in which so many people are obese. 

Referring to the hard choices, I conclude with the sobering thought that the cultural and psychosocial benefit-to-risk ratio of a major campaign to address obesity in the black community, and perhaps other communities as well, is not at all clear. Raising awareness and concern about obesity may render people in communities of color less satisfied with themselves and less able to cope with one more thing for which we cannot yet offer a good solution. This is a reason for serious reflection as we go forward.

*Adapted from the author's keynote lecture for the Charles C. Shepard Science Awards ceremony, Centers for Disease Control and Prevention and Agency for Toxic Substances and Disease Registry, Atlanta, Ga, June 21, 2004.*

## Figures and Tables

**Table:** Comparison of Crude Death Rates per 100,000 Person-Years, Associated Relative Risks (Rate Ratios), and Rate Differences Between African American and White Women in the Cancer Prevention Study II (CPS-II), by Body Mass Index (BMI)^a^

	**Death Rates**			**Rate Difference** ** by Weight Status^b^**		**Rate Ratio** ** by Weight Status^c^**	

**BMI Category**	**African American**	**White**	**Difference**	**African American**	**White**	**African American**	**White**
Normal (BMI 18.5–24.9)	935	677	258	ref	ref	ref	ref
Overweight (BMI 25.0–29.9)	946	767	179	11	89	1.1	1.1
Obese (BMI ≥30.0)	1146	1042	103	211	365	1.2	1.5

a Table adapted from Table 3 in Stevens J. Obesity and mortality in African-Americans. Nutr Rev 2000;58:346-53. Copyright 2000, International Life Sciences Institute.

b Death rate minus the rate in the referent group (ref), within ethnicity.

c Ratio in comparison to the referent group (ref), within ethnicity; calculated from data in source table.
